# Achalasia as an Unusual Cause of Acute Cellular Rejection of a Transplanted Heart

**DOI:** 10.1155/2022/2054727

**Published:** 2022-09-28

**Authors:** Amanda Fernandes, Crystal Lihong Yan, Phillip Ruiz, Nina Thakkar Rivera

**Affiliations:** ^1^Division of Internal Medicine, University of Miami/Jackson Memorial Hospital, Miami, FL, USA; ^2^Departments of Surgery and Pathology, University of Miami/Jackson Memorial Hospital, Miami, FL, USA; ^3^Division of Cardiology, Miami Transplant Institute, University of Miami/Jackson Memorial Hospital, Miami, FL, USA

## Abstract

A 68-year-old female with end-stage heart failure presented to the hospital for heart transplant. She was diagnosed with achalasia 14 months prior and treated with frequent botulinum toxin injections. She underwent orthotopic heart transplant on the day of admission and was extubated a few days later. She developed intractable nausea and vomiting. Her first endomyocardial biopsy revealed moderate, approaching severe rejection. She was treated with high-dose intravenous pulse steroids. Fluoroscopy at the time of follow-up biopsy showed undigested pills in her esophagus with narrowing at the distal end and thus failure to deliver immunosuppressive therapy. This case highlights achalasia as an etiology for acute rejection and its potential management.

## 1. Introduction

Acute allograft rejection is the most feared complication following heart transplantation. There are several types of rejection of which the classification and appropriate treatment are based on the type (cellular or humoral), timing posttransplant (hyperacute, acute, and chronic), and grade (mild, moderate, and severe) [1]. The most common type of heart transplant rejection is acute cellular rejection. It is a T-cell-mediated process that often happens in the first 3 to 6 months posttransplant. However, it may occur any time and can be prevented with adequate doses of maintenance immunosuppressive therapy. Hyperacute rejection is rare given the ABO matching between donor and recipient prior to transplant and the prospective cross-matching for HLA. In high-risk cases, higher doses of immunosuppression are given perioperatively (called induction therapy) to avoid the donor antigen expression and subsequent immune response by the recipient [1, 2].

While rejection is mostly commonly thought of as having an immunologic etiology, the etiology can also be mechanical. Esophageal dysmotility and subsequent failure to deliver immunosuppressive therapy are a rare etiology of allograft rejection posttransplant. Prompt identification of a mechanical etiology is crucial to treat rejection. In the following case, we report an unusual cause of transplant rejection due to a preexisting anatomic abnormality from achalasia resulting in the inability to achieve proper immunosuppression.

## 2. Case Presentation

A 68-year-old female with a past medical history of end-stage heart failure with reduced ejection fraction secondary to nonischemic cardiomyopathy on chronic milrinone infusion for the past 13 months, presented to the hospital at the instruction of her transplant team after being matched with a donor. She was originally listed as UNOS status 1B and later converted to UNOS status 4 with the new allocation system. She had also been diagnosed with achalasia 14 months prior with biopsies negative for eosinophilic esophagitis and serologies negative for Chagas disease. Due to high surgical risk, her achalasia was managed with frequent botulinum toxin (Botox) injections, resulting in improvement of her dysphagia. The last injection was one month prior to heart transplant.

**Figure 1:**
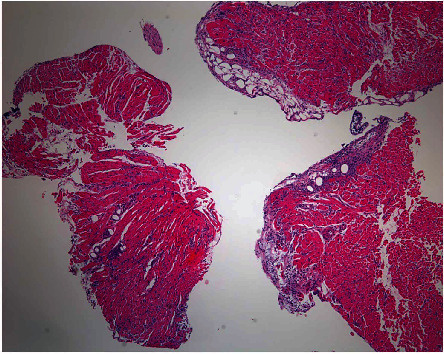
First endomyocardial biopsy posttransplant demonstrating moderate acute cellular rejection with mildly diffuse lymphocytic infiltrates and focal myocardial ischemic injury.

**Figure 2:**
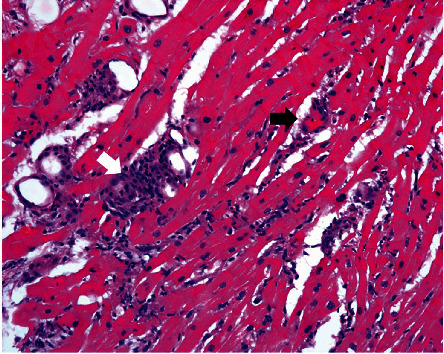
Close up image showing cardiac myocytes surrounded by dense collections of inflammatory cells (white arrow) and focal capillaritis (black arrow), consistent with grade 2R (borderline 3R) rejection.

**Figure 3:**
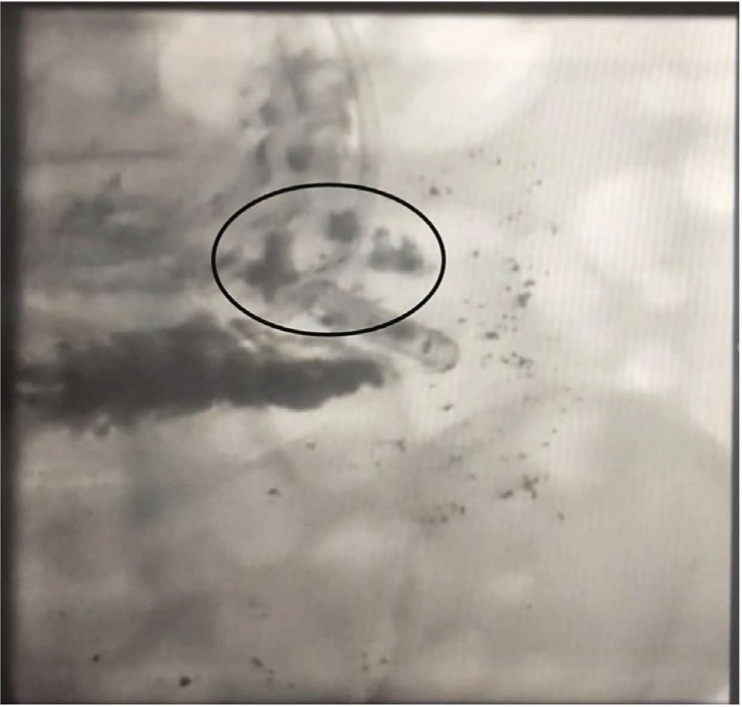
Fluoroscopy images at the time of endomyocardial biopsy demonstrating residual medications at the distal end of the esophagus (circle).

**Figure 4:**
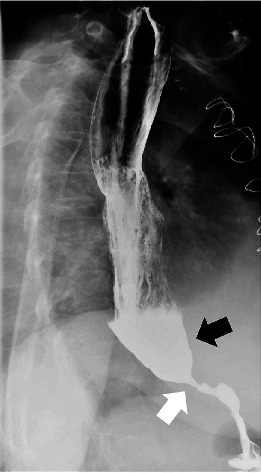
Barium swallow study demonstrating achalasia. There is significant narrowing at the lower esophageal sphincter (white arrow) with pooling of contrast in the distal esophagus (black arrow) and markedly delayed transit of contrast into the stomach.

She underwent orthotopic heart transplant on the day of admission and was successfully extubated on postoperative day 4 (POD). However, over the following days, she developed intractable nausea and vomiting. Her first endomyocardial biopsy on POD 12 revealed grade 2R approaching 3R rejection (Figures 1 and 2). She remained hemodynamically stable and was treated with high-dose intravenous pulse steroids. Fluoroscopy at the time of follow-up biopsy revealed undigested pills in her esophagus and narrowing at the distal end (Figure 3). This led to a barium swallow series (Figure 4), which confirmed the diagnosis of achalasia as the etiology for her acute rejection.

To address the cause of her rejection, she underwent percutaneous endoscopic gastrostomy (PEG) tube placement within three weeks of transplantation for the sole purpose of immunosuppression delivery. Furthermore, she received a temporizing injection of Botox to her lower esophageal sphincter with relief of her refractory nausea and vomiting. She was discharged after a 5-week hospital course in stable condition and with control of her acute rejection down to grade 1R. Unfortunately, her disease course was further complicated by multiple admissions for a clogged PEG tube, compromising her immunosuppression delivery. In addition, she continued to receive multiple Botox injections for dysphagia and severe acid reflux until it no longer worked. Given her history of multiple PEG tube malfunctions and inability to take her transplant antirejection medications orally, she underwent a laparoscopic Heller myotomy procedure 7 months posttransplant. Since then, she has remained without rejection and on stable doses of immunosuppression.

## 3. Discussion

Achalasia is rare type of neurodegenerative dysmotility disorder with an estimated incidence of around 1.5 to 2 per 100,000 person-years [3]. It affects the lower portion of the esophagus causing dysphagia, weight loss, chest pain, acid reflux, and food regurgitation [4]. The most definitive therapy for achalasia is surgical myotomy, followed by graded pneumatic dilation [5]. However, posttransplant, surgery is ideally avoided for up to 1 year.

In our case, we kept the patient stable without rejection for as long as we could and ultimately opted for surgical myotomy sooner than 1 year because of her risk for rejection without adequate delivery of her immunosuppressive medications. Botox can be a temporary treatment in patients with high surgical risk, as was our patient, although its efficacy is debatable. Studies have shown minimal improvement in symptoms for a limited time [4] or no improvement [6].

Solid organ transplant recipients are commonly affected by gastrointestinal complications, which can be related to the surgical procedure itself, opportunistic infections, or side effects from immunosuppressive medications [7]. In a retrospective cohort study, the presence of esophagogastric outflow obstruction, gastroesophageal reflux, and incomplete transit of food bolus were independently associated with obstructive chronic lung allograft dysfunction [8], but there is no data on those factors being related to heart transplant rejection.

To the best of our knowledge, this is the first reported case describing a patient with a pretransplant diagnosis of achalasia who was managed initially with Botox injections due to her high surgical risk and developed acute heart transplant rejection because of failure to deliver an immunosuppressive regimen at the absorbing portion of her gastrointestinal tract. Fluoroscopy performed during follow-up endomyocardial biopsy revealed the undigested pills in her distal esophagus and was key in allowing prompt recognition of the true etiology behind her acute rejection.

The current indications for PEG tube placement are for long-term enteral feeding in patients with functional gastrointestinal systems or for gastric decompression [9]. But here, we report another feasible and life-saving indication, which is to avoid transplant rejection in patients with esophageal dysmotility. In our case, the PEG tube was a temporary measure until her recovery post-heart transplant in order to receive the appropriate long-term solution.

## 4. Conclusion

We described a case of achalasia causing acute allograft rejection due to the anatomic inability to deliver immunosuppressive therapy after heart transplantation. In patients undergoing evaluation for solid organ transplant and with clinical concern for esophageal dysmotility, it is important to rule out achalasia on barium swallow and manometry studies. If confirmed, a multidisciplinary plan should be created prior to transplantation given the heightened risk of acute allograft failure.
